# FRET-Based Analysis of AgInS_2_/ZnAgInS/ZnS Quantum Dot Recombination Dynamics

**DOI:** 10.3390/nano10122455

**Published:** 2020-12-08

**Authors:** Maksim Miropoltsev, Vera Kuznetsova, Anton Tkach, Sergei Cherevkov, Anastasiia Sokolova, Viktoria Osipova, Yulia Gromova, Mikhail Baranov, Anatoly Fedorov, Yurii Gun’ko, Alexander Baranov

**Affiliations:** 1 Center of Information Optical Technology, ITMO University, 197101 Saint Petersburg, Russia; miropoltsev_m@niuitmo.ru (M.M.); aptkach@itmo.ru (A.T.); s.cherevkov@itmo.ru (S.C.); avsokolova@itmo.ru (A.S.); vaosipova@itmo.ru (V.O.); 193347@itmo.ru (Y.G.); mbaranov@itmo.ru (M.B.); a_v_fedorov@itmo.ru (A.F.); a_v_baranov@itmo.ru (A.B.); 2 Chemistry School, Trinity College Dublin, Dublin 2 Dublin, Ireland; igounko@tcd.ie

**Keywords:** AgInS_2_, FRET, ternary quantum dots, cyanine dyes, time-resolved fluorescent spectroscopy, donor-acceptor pair recombination, core/shell nanostructures

## Abstract

Ternary quantum dots (QDs) are very promising nanomaterials with a range of potential applications in photovoltaics, light-emitting devices, and biomedicine. Despite quite intensive studies of ternary QDs over the last years, the specific relaxation channels involved in their emission mechanisms are still poorly understood, particularly in the corresponding core-shell nanostructures. In the present work, we have studied the recombination pathways of AgInS_2_ QDs stabilized with the ZnAgInS alloy layer and the ZnS shell (AIS/ZAIS/ZnS QDs) using time-resolved fluorescence spectroscopy. We have also investigated FRET in complexes of AIS/ZAIS/ZnS QDs and cyanine dyes with the absorption bands overlapping in the different regions of the QD emission spectrum, which allowed us to selectively quench the radiative transitions of the QDs. Our studies have demonstrated that FRET from QDs to dyes results in decreasing of all QD PL decay components with the shortest lifetime decreasing the most and the longest one decreasing the least. This research presents important approaches for the investigation of ternary QD luminescence mechanisms by the selective quenching of recombination pathways. These studies are also essential for potential applications of ternary QDs in photodynamic therapy, multiplex analysis, and time-resolved FRET sensing.

## 1. Introduction

Ternary quantum dots (QDs) are semiconductor nanocrystals based on compounds containing elements from I–III–VI_2_ groups. The most popular materials, namely AgInS_2_ (AIS) and CuInS_2_ (CIS), gained much attention during the last decade as non-toxic vis- and NIR-emitters with extended tunability of their photophysical parameters [1,2,3,4,5,6,7]. Unlike classical binary Cd- and InP-based systems, ternary QDs are highly tolerant to off-stoichiometry and crystallographic defects both in the volume and on the surface of nanocrystals [8,9,10,11,12,13]. The composition and structure strongly affect ternary QD optical properties [14,15,16,17,18]; the latter may be, therefore, tuned in a broad range by changing the stoichiometry, doping, and alloying with the shell material during the synthesis or via post-synthetic treatments [3,19,20,21,22].

Due to their low toxicity and unique photoluminescence (PL) properties, ternary nanocrystals have become particularly attractive for the energy conversion applications [23,24]. High absorption coefficients, broad tunable bandwidths and large Stokes shifts in combination with easily scalable and cost-effective production have made them promising candidates for the next-generation solar cells and solar concentrators [25,26,27,28,29], light-emitting devices [30,31,32], and photocatalysts [33,34,35]. Furthermore, AIS and CIS QDs are actively replacing conventional Cd-based nanocrystals in biomedicine-related applications, such as bioimaging, as has been recently shown by several groups [36,37,38,39,40,41]. Another important subclass of applications in life sciences employs ternary QDs as energy donors in the complexes with Förster resonant energy transfer (FRET) [41,42]. These complexes might be used for photodynamic therapy [43,44,45] and assays for the multiplex analysis [46,47,48].

The main reason why ternary QDs are so attractive for the FRET-based applications is their characteristic emission. Their PL FWHM is typically in the range of 100–400 meV, which is much broader than in the case of binary nanocrystals [1,2,3,6]. Such broad bands favor versatility of the energy donor, since more acceptors with different absorption bands are likely to overlap with the donor’s emission. Moreover, PL lifetimes of ternary QDs are distributed along the emission band with the shorter times at higher energies and the average values of hundreds of nanoseconds [10,12,49,50,51]. Such long wavelength-dependent lifetimes are advantageous for two reasons: (a) they help to separate the short-lived autofluorescence of the biological surroundings, thereby increasing the sensitivity of a sensor [46]; (b) they allow for lifetime multiplexing—a technique which implies barcoding of luminescent labels based on their lifetimes rather that PL peak positions [47,48,52,53,54].

Substantial effort has been put into the study of ternary QD photoluminescence, but the main mechanisms behind it are still unclear. Such models as donor-acceptor pair recombination (DAP) [9,10,11,12,13,14], free-to-bound recombination [21,50,55,56,57,58,59,60,61] and exciton self-trapping [17,49,51,62,63,64] have been proposed, each of them explaining, but only partially, the experimentally observed properties like broadband emission with hundred-nanosecond wavelength-dependent lifetimes [1,6,7]. Additional complexity arises from the fact that both AIS and CIS quantum dots have been reported to have two to four exponential components in the photoluminescence decay. Different research groups have been speculating whether these decay components may be interpreted in terms of specific recombination pathways, or they are merely a rough approximation to the (likely) continuous lifetime distribution in the QD ensemble [8,12,16,18,29,32,42,55,65,66,67,68].

Thus, the lack of knowledge regarding the nature of AIS and CIS emission hampers further development of their practical applications, including the corresponding FRET-based products in the life sciences area. On the other hand, recent studies show that FRET may be also utilized as an effective tool to analyze the mechanisms of ternary QD recombination [42]. Therefore, here we have developed an experimental approach to study the luminescence mechanisms of AgInS_2_ QDs stabilized with the ZnAgInS alloy layer and the ZnS shell (AIS/ZAIS/ZnS QDs) using time-resolved fluorescence spectroscopy technique. Cyanine dyes absorbing in different regions of the QD luminescence spectrum were used to perform the selective energy transfer from different energy states of the QDs. This allowed us to obtain unique information about the relaxation channels involved in the emission of the ternary QDs.

## 2. Materials and Methods

### 2.1. Materials

1-Dodecanthiol (DDT, 98%) was obtained from Acros Organics; Zinc stearate (65%), Zinc oxide (99%) and Indium (III) acetate (In(Ac)_3_, 98%), Silver (I) nitrate (AgNO_3_, ≥99.5%), Oleic acid (OA, 90%), 1-Octadecene (ODE, 90%), Oleylamine (OlAm, 70%), 2-Ethylhexanoic acid (99%), Thiourea (99%), Triethyleneglycol dimethylether (98%), L-cysteine hydrochloride monohydrate (98%), Potassium hydroxide (KOH, 90%) were purchased from Sigma Aldrich (Saint Petersburg, Russian Federation). Organic cyanine dyes: 3,3′-diethylthiacarbocyanine iodide (Cy3) (95%) and 3,3′-diethylthiadicarbocyanine iodide (Cy5) (98%) were purchased from Sigma-Aldrich. Solvents: Isopropanol (99.8%), acetone (99.75%), and chloroform (99.9%) were obtained from Vekton (Saint Petersburg, Russian Federation). All chemicals were used without further purification. Ultrapure water (Milli-Q) was used throughout the experiments.

### 2.2. Synthesis of AIS/ZAIS/ZnS QDs

**Synthesis of AgInS_2_ cores.** QDs were synthesized according to the modified method [69] described in details by Kuznetsova et al. [48]. Briefly, AgNO_3_, In(Ac)_3_, DDT and OA were added to a three-necked flask and degassed with subsequent heating up to 185 °C under the inert atmosphere. The flask was maintained at this temperature until the solution turned bright red. The growth stage was stopped by ODE injection. Afterwards, the reaction solution was heated up to 210 °C for first shell growing.

**ZnS shell growing.** The ZnS shell was formed in two steps. The first (inner) ZnS shell growing procedure which led to the formation of the ZAIS alloy layer was performed using zinc stearate previously dissolved in ODE. The solution was degassed and heated up to 160 °C before the injections. 2.5 mL of the obtained Zn precursor was injected into the preheated flask with the QD cores mixture; injections (0.5 mL) were repeated every 15 min. Total time of the first shell growing stage was 45 min at 210 °C. After that, the flask was cooled down and the size-selective precipitation with acetone was performed. QDs of all sizes were redissolved in toluene, and only the green AIS/ZAIS fraction was used for the second shell growth.

The second (outer) ZnS shell layer was applied using the Zn and S precursors synthesized in advance [22]. Briefly, the zinc precursor was prepared by mixing 2-ethylhexanoic acid, zinc oxide, and ODE with subsequent heating until a clear solution was obtained. For the S precursor, thiourea and triethyleneglycol dimethylether were mixed with sonication. Afterwards, both precursors were injected dropwise by portions at 180 °C within 1 h in the predegassed and preheated mixture of the QD solution in toluene, ODE, and OlAm. The obtained green QDs with the double-shell were precipitated with acetone and redissolved in chloroform for the further water solubilization.

### 2.3. Solubilization of AIS/ZAIS/ZnS QDs and Formation of the QD-Dye Complexes

The QDs were solubilized by the procedure described in [22]. Briefly, AIS/ZAIS/ZnS QDs were additionally washed twice with isopropanol and acetone with subsequent dissolution in chloroform to remove the excess of the original ligand. Next, L-cysteine hydrochloride monohydrate solution in methanol was added, and the QD mixture was stirred for 5 min and purified twice using methanol. The obtained precipitate was dissolved in 0.01 M aqueous solution of KOH. The QD concentration in the used stock solution was determined by thermogravimetric analysis to 2.5 × 10^–7^ M.

The QD-dye complexes were prepared electrostatically by mixing the negatively charged QDs with the positively charged dye molecules Cy3 and Cy5 at the dye:QD ratio of 2:1. This ratio was used to minimize the amount of QDs without the dye molecules on the surface, assuming that they obey a Poisson distribution. Larger ratio, however, would result in the strong quenching of dyes in the complex, as has been previously shown for the similar system [48].

### 2.4. Equipment

The UV-VIS absorption spectra were recorded using a UV-Probe 3600 spectrophotometer (Shimadzu, Kyoto, Japan). The steady-state PL spectra were obtained with Cary Eclipse spectrofluorometer (Agilent, Santa Clara, CA, USA). Time-resolved fluorescence spectroscopy measurements were performed using a time-correlated single photon counting fluorescence microscope MicroTime 100 (PicoQuant, Inc., Berlin, Germany.) equipped with a 405 nm pulsed laser LDH-P-C-405B (PicoQuant, Inc., Berlin, Germany) with pulse duration of 20 ps. Different detection wavelengths were selected with a holographic bandpass filter with the 10 nm bandwidth tunable in the spectral range of 430–780 nm. Laser frequency was adjusted using an additional signal generator SFG-71003 (Good Will Instek, Montclair, CA, USA). STEM images of the AgInS_2_ core QDs and the AIS/ZAIS/ZnS QDs were measured with a Scanning Electron Microscope Merlin (Zeiss, Oberkochen, Germany) operated at 30 kV on copper grids with ultrathin carbon films. Thermogravimetric analysis was performed on a STA 429 CD Simultaneous thermal analyzer (Netzsch-Gerätebau GmbH, Selb, Germany) using a platinum-platinum rhodium holder for TG + DSC samples.

## 3. Results and Discussion

### 3.1. Spectroscopy of the Free AIS/ZAIS/ZnS QDs and the QD-Dye Complexes

**Figure 1:**
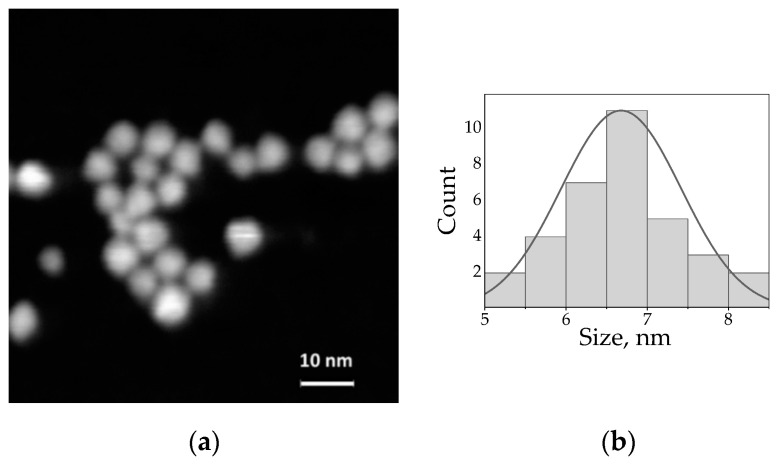
(**a**) STEM image of the water-soluble AIS/ZAIS/ZnS QDs; (**b**) Size distribution of the AIS/ZAIS/ZnS QDs (the mean diameter is 6.7 ± 0.8 nm).

Water-soluble negatively-charged AIS/ZAIS/ZnS quantum dots, which we used throughout the work, were prepared in three steps, as was described in [22,69]: (1) synthesis of AIS cores in the organic solvent; (2) two-step shell growth; (3) ligand exchange with L-cysteine. According to the literature, ZnS shell growth over the ternary QD cores may lead to partial alloying of the original particles [12,70]. Therefore, we describe the inner ZnS shell in terms of the ZAIS alloy layer, while the additional outer ZnS shell eliminates surface defects and ensures localization of the electron-hole pair in the QD core. As a result, ligand exchange does not significantly change the PL QY. STEM analysis of the QDs (Figure 1) revealed that the average size of AIS/ZAIS/ZnS QDs is 6.7 ± 0.8 nm after the growth of the double-shell.

QD-dye complexes for FRET studies were formed in aqueous solutions via an electrostatic interaction between negatively charged ligand molecules on the QD surface and positively charged cyanine dyes at the dye:QD ratio of 2:1. Assuming that the number of dye molecules per quantum dot obeys a Poisson distribution, the 2:1 ratio is needed to minimize the amount of QDs which bare no acceptor at all; on the other hand, larger ratio would result in the strong quenching of dyes in the complex due to the formation of molecular dimers, as has been previously shown for the similar system [48].

**Figure 2:**
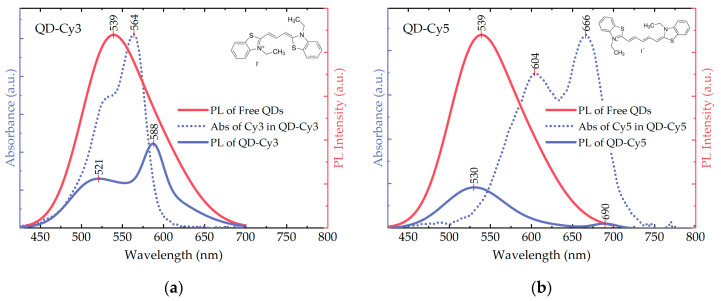
(**a**)—QD-Cy3, (**b**)—QD-Cy5. Spectral overlap of the free AIS/ZAIS/ZnS QD PL (red solid curves) with the dye absorbance in the respective QD-dye complexes (blue dotted curves). The dye absorbance was obtained by subtraction of the QD absorbance from the complex absorbance. Photoluminescence of the QD-dye complexes (blue solid curves) indicate the energy transfer from the QDs to the dyes (see text for the details). Insets: structural formulae of Cy3 (**a**) and Cy5 (**b**).

In Figure 2, photoluminescence of the free QDs and the QD-dye complexes is presented, together with absorbance of the dyes in the complexes. In line with the previous reports [1,2,3,4,5,6,7], QDs exhibit a broad emission band with the peak at 539 nm and FWHM of 108 nm. The PL QY of the three QDs was estimated to be 6%. Here and further, the 405 nm excitation was used for the PL measurements of the QDs and the QD-dye complexes. The dye absorbance curves (blue dotted lines in Figure 2) were obtained by subtraction of the QD absorbance from the complex absorbance. PL peaks of Cy3 and Cy5, as well as their absorption bands, exhibit the redshift when the dye molecule is bound to the QD surface. This effect is well-known in the literature and considered as an indicator of the successful complex formation [48,71]. In the complexes, the low-energy absorption (PL) bands of Cy3 and Cy5 cyanines have maxima at 564 (588) nm and 604/666 (690) nm, respectively.

We have selected the dyes with the absorbance overlapping with the PL spectrum of QDs in different spectral regions, that allowed us to achieve FRET from different energy states separately. Since Cy3 and Cy5 cyanines cannot be directly excited by the 405 nm radiation, their PL peaks at this excitation wavelength indicate the presence of the energy transfer (Figure 2, blue solid curves). As donors, QDs donate their energy from that part of the spectrum which overlaps with the acceptor absorption band. Both Cy3 and Cy5 in complexes have absorption peaks at lower energies than the original QD PL maximum. Therefore, upon the energy transfer, the QD PL components in the complex PL curves exhibit the blueshift in comparison to the free QD emission [42]. Cy5, absorbing at lower energies than Cy3, leads to a less pronounced blueshift (≈10 nm and ≈20 nm, respectively).

### 3.2. Analysis of AIS/ZAIS/ZnS QD Recombination Dynamics

**Figure 3:**
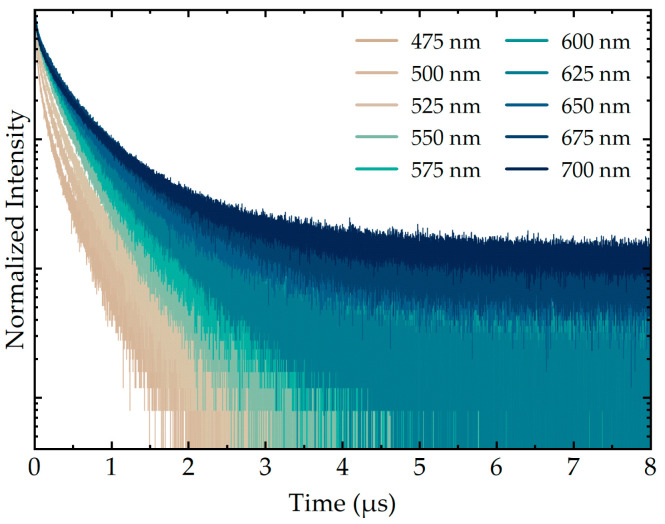
Normalized PL decay curves of the free AIS/ZAIS/ZnS quantum dots measured along the QD emission band with a 10 nm bandpass filter.

AIS/ZAIS/ZnS time-resolved emission spectroscopy measurements were performed at different detection wavelengths using a holographic bandpass filter with the 10 nm bandwidth tunable in the spectral range of 430–700 nm. Normalized PL decay curves of the free QDs are shown in Figure 3. The lifetimes of QDs increased with the wavelength, and similar behavior of ternary QDs was observed in other studies [1,6,7]. This effect is typically explained in terms of the donor-acceptor pair recombination theory [8,66,68]. Donor states below the conduction band are usually created by S vacancies and interstitial Ag atoms, while Ag vacancies and interstitial S atoms can form acceptor levels above the valence band [8,72]. Besides, surface defects of QDs, such as vacancies, dangling bonds, and oxygen adatoms, can also take part in the DAP recombination, providing local sites for the carrier relaxation [10,12]. Due to Coulombic interactions, more closely localized donor-acceptor pairs have higher energy than the distant ones. The recombination probability between the electrons and holes on close pairs is also higher because of larger overlap of their wavefunctions. Thus, high-energy transitions occur faster, which leads to shorter decay times for higher emission energies.

The acquired decay curves of AIS/ZAIS/ZnS QDs (Figure 3) are not single-exponential, which is also common for this type of nanocrystals [1,6,7]. The explanation of the multiexponential nature of ternary QD emission, as well as the number of the exponents, is still under debate. There are several theories regarding the origin of the responsible recombination channels. In different reports, the decay curves of ternary QDs were fitted with two [8,16,29,32,55], three [12,18,65,66,67,68], or even four [42] exponents. In the case of two exponents, the longer one is usually attributed to the DAP recombination inside the QD core, while the shorter one is associated with the surface defects [10]. The appearance of the third exponent was explained in different ways. Several research groups advocate the idea of Liu et al. that the free-to-bound mechanism involving the sub-bandgap defect states gives rise to the additional decay component [18,66,68]. Alternative explanation, which was initially proposed by Komarala et al., suggests that the longest and the middle lifetime components correspond to the DAP of the nearest neighbors and between the pairs of the next closest neighbors, respectively [12,65,67].

To improve the luminescent properties of the QDs, a wide-bandgap material shell, most commonly ZnS, can be grown to localize the exciton in the QD core. It has been shown, that the growth of the shell reduces the overall contribution of surface defects to the ternary QD luminescence, and consequently the contribution of the shorter lifetime component to the total decay curve [10,65,70]. Moreover, the small lattice mismatch between AIS and ZnS crystal structure favors the interdiffusion of their components, what leads to formation of a 2–3 monolayer ZnAgInS (ZAIS) alloy with the variable composition at the junction with the core [14,70].

In addition, many studies have shown that the variations in the ternary QD stoichiometric ratios lead to a dramatic change in the lifetimes [18,73,74]. For example, in the paper by Xiang et al., the average PL lifetime decreased from 588 to 303 ns as the ratio of Ag:In decreased from 1:1 to 1:6 [74]. In our case, zinc primarily replaces silver when embedded into the AIS lattice, so the Ag:In ratio decreases. Despite the AIS and ZnS structures are close, they still differ in lattice parameters by several percent [70]. As a result, structural stresses are created at the interface, that can also lead to new recombination channels and variations in the decay times.

In the present work, AIS/ZAIS/ZnS PL decay curves were fitted using a three-exponential model:

(1) I(t)=A0+A1e−tτ1+A2e−tτ2+A3e−tτ3,

where $A_{0}$ is the background noise level, $A_{1}$, $A_{2}$ and $A_{3}$ are the amplitudes and $\tau_{1}$, $\tau_{2}$ and $\tau_{3}$ are the decay times of the first, second and third exponent, respectively. The three-exponential model was assumed since it provided the acceptable fitting results (χ^2^ < 1.1) with the lowest possible number of components. Apart from the lifetimes, the relative contribution of each recombination pathway to the overall QD emission was calculated according to the formula:

(2) $$C_{i}\left[\%\right]=\frac{A_{i}\tau_{i}}{\sum_{i=0}^{3}A_{i}\tau_{i}}\times 100\%.$$

Prior to further discussion, let us address the concept of multiexponential decay in more detail. When several components are seen in a decay curve, they are usually interpreted as separate fluorescent entities, each with its own characteristic lifetime. In the case of ternary QDs, the presence of three distinguishable lifetimes suggests that the three recombination pathways coexist in the ensemble. Given the complexity of ternary materials, it is likely that these pathways are related to the different types of structural defects that provide local relaxation sites for the charge carriers. Both the concentration and the relaxation rate may differ for these defects, resulting in different probability for a quantum dot to emit via one of the pathways. Furthermore, particle-to-particle variations are also possible. The number of emission events for each channel depends on the state population and the recombination probability. To account for the number of emission events via each channel, we use the relative contributions, Equation (2). The latter, in fact, have the meaning of a quantum efficiency, that is, the number of photons with the given lifetime to the total number of photons emitted at a certain wavelength.

**Figure 4:**
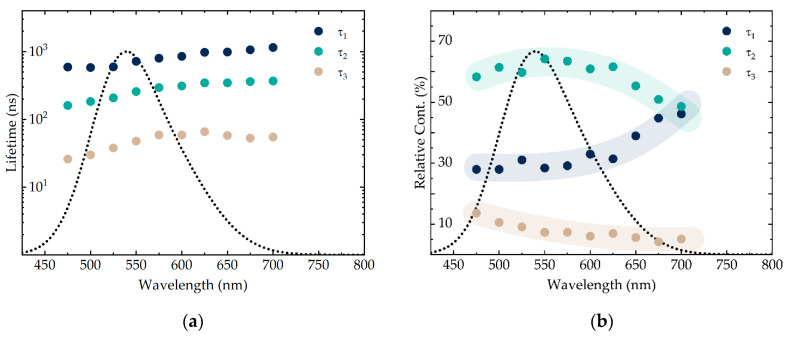
The PL decay components $\tau_{1}$, $\tau_{2}$ and $\tau_{3}$ (**a**) and their relative contributions (**b**) acquired calculated from the decay curves of the free AIS/ZAIS/ZnS quantum dots. The semi-transparent colored lines in (**b**) indicate the trend.

The calculated lifetimes and the relative contributions for the free AIS/ZAIS/ZnS QDs are presented in Figure 4. All three lifetime components increase with the wavelength. The shortest lifetime ($\tau_{3}$), which is usually attributed to the surface defect-related emission [10,12,65,70], has the smallest contribution of 5–10%. Since our QDs were covered with the double-shell, it is reasonable that the surface defect-related component did not contribute significantly to the overall emission. In addition, surface defects are shallow, i.e., they mostly emit at higher energies. In our case, the contribution of $\tau_{3}$ also decreases with the wavelength (Figure 4b, sandy trend line). The middle lifetime ($\tau_{2}$) with the largest contribution (up to ≈65%) is presumably related to the ZAIS alloy layer, whereas the longest lifetime ($\tau_{1}$) can be assigned to the DAP recombination within the nanocrystal core [12,65]. Due to the non-stoichiometry and structural distortions, it is likely that the concentration of defects is higher in the intermediate alloy layer than in the core of the QDs. As a result, the contribution (i.e., the relative number of photons emitted) of the alloy-related recombination channel is higher than of the core-related. Moreover, alloys with higher zinc content emit light at higher energies than the pure AIS [70], so the relative contribution of $\tau_{2}$ decreases towards the end of the spectrum, while $\tau_{1}$ increases (Figure 4b, cyan and dark blue trend lines, respectively). Notably, both the lifetimes and the relative contributions of our AIS/ZAIS/ZnS QDs are essentially similar to that of quaternary ZnAgInS QDs, according to the publication by Chevallier et al. [12]. This fact may suggest that the mechanisms responsible for the emission of quaternary and core/shell ternary quantum dots are also close by the nature.

### 3.3. FRET from AIS/ZAIS/ZnS QDs to the Dyes in the QD-Dye Complexes

QD-dye complexes were prepared by the means of an electrostatic interaction in the aqueous solutions. Upon the complex formation, time-resolved fluorescence spectroscopy measurements were conducted to address the changes in the QD recombination dynamics. The excitation wavelength was again 405 nm, and the emission was collected in the same way as for the free QD sample. The fitting procedure was conducted as follows. For those wavelengths where only the QDs emit, the decay curves were successfully fitted with the three-exponential model, Equation (1). In those regions where the QD emission overlaps with the sensitized acceptor emission, the appropriate fitting model depended on the sample. For the QD-Cy3 complex, starting from 575 nm, the acceptable fitting results could be obtained by adding the fourth component. In the case of QD-Cy5, no additional components were obtained. We would like to emphasize that the number of exponents in the decay fitting model does not necessarily reflect the actual number of the recombination channels, implying the complexity of the system and the ambiguity of the fitting procedure. Nevertheless, the acquired data can still provide useful information regarding the photoluminescence of the QD-dye complexes.

**Figure 5:**
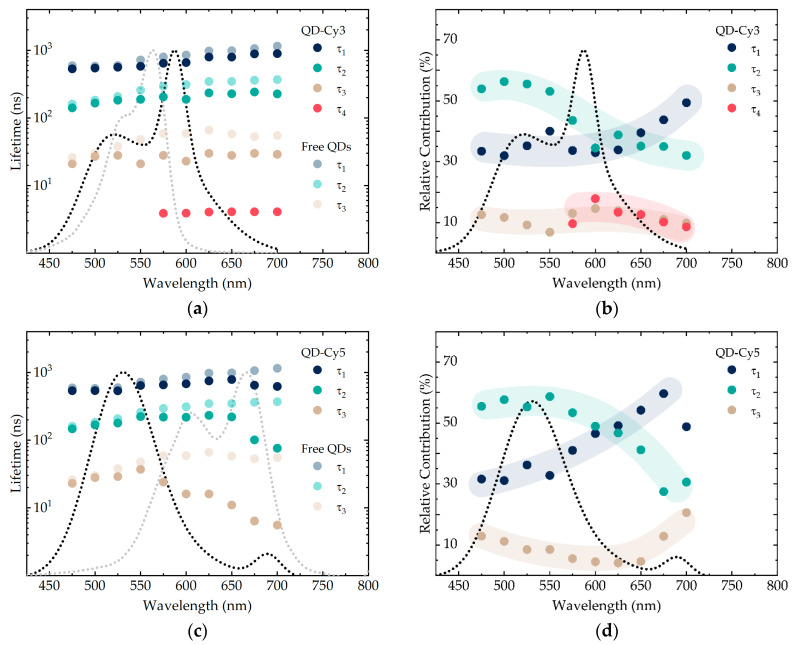
The PL decay components $\tau_{1}$, $\tau_{2}$ and $\tau_{3}$ (**a**,**c**) and their relative contributions (**b**,**d**) for the complexes of AIS/ZAIS/ZnS quantum dots and Cy3 and Cy5 dyes. The PL (black dotted lines) and the absorbance (gray dotted lines) spectra of the corresponding complexes are shown for convenience. The semi-transparent colored lines in (**b**,**d**) indicate the trend.

The lifetimes and the relative contributions calculated for QD-Cy3 and QD-Cy5, along with the absorbance and PL spectra of the corresponding complexes, are presented in Figure 5. After formation of the complexes, all three donor lifetimes decrease indicating that FRET affects all recombination channels of AIS/ZAIS/ZnS QDs. Interestingly, in the case of Cy3, the QD lifetimes also drop beyond the region of the dye absorption, i.e., above 600 nm. This may suggest that, in ternary QDs, the relaxation takes place from the states emitting at higher energies to the states emitting at lower energies, which is consistent with the reports stating that ternary QDs, in contrast to binary QDs, possess inherently broad PL bands [51,64].

QD decay components primarily drop in the region of the dye absorption. Thus, the decrease may be attributed to the resonance energy transfer where the energy is transferred from every channel. The relative decrease of the PL lifetime can be used to calculate the FRET efficiency, according to the formula:

(3) $$E_{FRET}=\frac{\tau_{D}-\;\tau_{DA}}{\tau_{D}}=\frac{R_{0}^{6}}{R_{0}^{6}+R^{6}}.$$

where $\tau_{D}$ and $\tau_{DA}$ are the lifetimes of the donor in the absence and the presence of the acceptor, respectively, $R_{0}$ is the Förster radius and R is the donor–acceptor distance. This formula, however, implies that no additional non-radiative relaxation channels are created upon the interaction between the donor and the acceptor. We do realize that such channels usually appear in the QD-dye complexes, although here we assume that only FRET is responsible for the drop in the AIS/ZAIS/ZnS QD decay times. The experimental FRET efficiencies calculated by Equation (3) from the data in Figure 5a,c are: ≈20% for $\tau_{1}$, 30–40% for $\tau_{2}$, and >50% for $\tau_{3}$, respectively. The calculations show that the lifetimes drop unequally, i.e., the corresponding channels have different FRET efficiency. With both Cy3 and Cy5, the shortest QD lifetime decreases the most, whereas the longest decreases the least. We would like to acknowledge that the calculated values must be treated cautiously and can merely be used for the qualitative analysis. To estimate the actual energy transfer efficiency, one should account for any new quenching pathways and the acceptor emission which is mixed with the donor PL in certain regions (Figure 5a,c). Such a thorough analysis is also interesting, although does not comply with the goals of this work.

Let us consider the three recombination channels of the quantum dots as separate donors for the energy transfer. Given that these three donors stem from the same sample in the same environment, the only parameters which may potentially alter the FRET efficiency are the channel quantum yield and the donor-acceptor separation. According to Förster theory, an increase in the donor quantum yield leads to an increase in the Förster radius (and, consequently, the FRET efficiency). In the experiment, higher FRET efficiency was observed for shorter lifetimes. This suggests that the multiexponential decay of AIS/ZAIS/ZnS QDs mainly arises from the variation in the radiative rather than the non-radiative recombination rate. Indeed, if the non-radiative rate was responsible for the multichannel emission, shorter lifetimes would mean lower channel quantum yields and hence lower FRET efficiencies, which contradicts our observations. We therefore agree with Xia et al. who came to the similar conclusion when studying the resonance energy transfer from CIS/ZnS QDs to the dark quenchers [42].

Another parameter which may result in the FRET efficiency variation is the distance between the donor and the acceptor. The established concept of defect-related emission of ternary quantum dots implies that the localization sites responsible for different emission pathways are spatially separated. In agreement with the literature, we attribute the longest lifetime to the QD core [12,65]. Since this type of recombination on average occurs the farthest from the surface-bound dye, it is reasonable that the corresponding FRET process is the least efficient. Accordingly, the shortest-lifetime component, which is possibly related to the QD surface, exhibits the energy transfer with the highest efficiency. The similar idea was invoked in the publication by Chong et al. who studied FRET from the streptavidin-conjugated CdTe quantum dots to the organic dyes [75]. We consider both explanations acceptable and presume that both the quantum yield and the defect location affect the efficiency of FRET from a particular quantum dot emission channel.

For the FRET-based applications, such as time-resolved imaging and lifetime multiplexing, the sensitized acceptor emission is of high importance. Unfortunately, it is often poorly characterized in the literature, and the main discussion is limited to the quenching of the donor states. In our case, all three quantum dot decay components decrease in the presence of the dyes, so it is intuitive to assume the three separate FRET processes and hence the three sensitized acceptor lifetimes. In experiment, however, for those wavelengths where the dyes emit, the fitting criterion (χ^2^ < 1.1) was already met when adding only one new ≈ 4 ns decay component for QD-Cy3 (Figure 5a) and no extra decay components for QD-Cy5 (Figure 5c). The latter may be due to the mixing of the dye lifetimes with each other and with the donor that emits at the same wavelength. The sharp drop in the QD decay that is seen in the region of Cy5 emission in Figure 5c suggests mixing of the dye lifetimes with the QD lifetimes and thus proves existence of the sensitized PL. It is also important to note that the free Cy3 and Cy5 characteristic lifetimes are ≈0.5 ns, which is an order of magnitude less than the resolved Cy5 sensitized emission [48]. This also indicates that the observed decay is related to FRET and not the direct excitation.

The acceptor sensitized emission can further be characterized by considering the variation in the quantum dot PL decay contributions. For the QD-dye complexes, the contributions are presented in Figure 5b,d. Since the contributions represent the fraction of the photons emitted with a certain lifetime, we can only talk about their change relative to each other. Interestingly, in comparison to the free QD sample (Figure 4b), $\tau_{2}$ contribution exhibits a pronounced decrease both in the case of QD-Cy3 and QD-Cy5. In other words, the number of photons from this recombination channel decreases to a higher extent than from the other two, whereas its FRET efficiency is not the highest. We presume that the dye sensitized PL, which was resolved for the QD-Cy5 complex, mainly arises from this quantum dot emission pathway. Originally, the middle lifetime channel had the highest contribution to the sample emission. Thus, it is intuitive that the corresponding FRET process will yield the highest intensity, which agrees with our considerations.

## 4. Conclusions

In the present report, we have investigated the recombination pathways of ternary AIS/ZAIS/ZnS core-shell QDs and studied FRET in complexes of AIS/ZAIS/ZnS QDs with cyanine dyes using time-resolved fluorescence spectroscopy. We have selected the dyes with the absorbance overlapping with the PL spectrum of QDs in different spectral regions enabling us to achieve the energy transfer from different relaxation channels separately. We use the three-exponential model to fit the PL decay curves. Our studies show that FRET in the complexes of QDs with cyanine dyes results in decreasing of all components of QD PL decay. Each QD energy relaxation channel was considered as a separate donor for FRET, and we used selective quenching of transitions to study the recombination dynamics of AIS/ZAIS/ZnS QDs. It was found that the shortest lifetime decreases the most, while the longest one decreases the least. The obtained results suggest that both the channel quantum yield and the defect location can alter the FRET efficiency. Our data is consistent with the model stating that the shortest component corresponds to the surface defects, and the longest—to the QD core. We have also analyzed the sensitized acceptor emission and conclude that the transferred energy is primarily coming from the QD alloy layer with the high concentration of defects. We believe that this research presents interesting approaches for the investigation of ternary QDs luminescence mechanisms by utilizing FRET to selectively quench the QD recombination pathways. These studies are also very important for potential applications of ternary QDs in photodynamic therapy, multiplex analysis, and time-resolved FRET sensing.
